# The effect of image position on the Independent Components of natural binocular images

**DOI:** 10.1038/s41598-017-18460-1

**Published:** 2018-01-11

**Authors:** David W. Hunter, Paul B. Hibbard

**Affiliations:** 1 Prifysgol Aberystwyth University, Department of Computer Science, Aberystwyth, SY23 3DB UK; 2 University of Essex, Department of Psychology, Colchester, CO4 3SQ UK

## Abstract

Human visual performance degrades substantially as the angular distance from the fovea increases. This decrease in performance is found for both binocular and monocular vision. Although analysis of the statistics of natural images has provided significant insights into human visual processing, little research has focused on the statistical content of binocular images at eccentric angles. We applied Independent Component Analysis to rectangular image patches cut from locations within binocular images corresponding to different degrees of eccentricity. The distribution of components learned from the varying locations was examined to determine how these distributions varied across eccentricity. We found a general trend towards a broader spread of horizontal and vertical position disparity tunings in eccentric regions compared to the fovea, with the horizontal spread more pronounced than the vertical spread. Eccentric locations above the centroid show a strong bias towards far-tuned components, eccentric locations below the centroid show a strong bias towards near-tuned components. These distributions exhibit substantial similarities with physiological measurements in V1, however in common with previous research we also observe important differences, in particular distributions of binocular phase disparity which do not match physiology.

## Introduction

It has long been known that visual acuity is greatest in the central portion of the visual field and decreases significantly towards the periphery^1,2^. This has been accounted for by the idea of a cortical magnification factor^3^, which holds that the number of neurons encoding a region of space decreases with eccentricity, while their receptive field size increases. This can account for performance on many low-level visual tasks, such as acuity^4,5^ and sensitivity to differences in orientation and frequency^6^. However, not all tasks follow this simple model^7^. Notably, stereoacuity decreases more rapidly with eccentricity than spatial acuity^8^.

The statistical properties of natural binocular images also vary with spatial location^9^. Distributions of physical disparities are biased towards crossed (near) disparities at the bottom of the image and uncrossed (far) disparities at the top^10^. The effects of spatial location on the natural distribution of disparities are of particular concern for the current study. These have been assessed recently^10^ for observers performing everyday tasks - walking indoors or outdoors, ordering coffee and making a sandwich. In this study, the disparities recorded during these tasks, weighted by the amount of time that Americans devote to these activities, were calculated as a function of image position.

These distributions showed a number of properties which are relevant to the current discussion. The median horizontal disparity had a clear vertical gradient, from crossed (near) disparities at the bottom of the image, through to uncrossed (far) disparities at the top. This pattern reflects both the presence of a ground plane^11–13^ and the fact that observers will have fixated on objects on a table-top while performing the tasks under consideration. The variation in horizontal disparity increased with increasing eccentricity. This again can be readily explained - as the distance from the centre of the image increases, points are less likely to be located on the fixated object, and thus more likely to be at a different distance^14^. The distributions of horizontal disparities were also skewed to have longer tails for uncrossed disparities, and were very highly peaked, more than has been predicted from geometrical assumptions in previous studies^14–16^.

Vertical disparities were much smaller than horizontal disparities. There was a consistent tendency to find positive values (points that project to a higher position in the left image than the right image) in the top left and bottom right quadrants. In contrast, negative vertical disparities tended to be found in the top right and bottom left quadrants. This pattern of results is consistent with geometrical predictions. Vertical disparities arise when an object is closer to one eye than the other, and thus projects a larger image in that eye. Thus, an object that is centred at eye-height, but to the left of an observer, will produce negative disparities in the bottom half of the image, and positive disparities in the top half. The standard deviation of vertical disparity increased away from the horizontal meridian, again in a way that follows from geometrical considerations; when the eyes are torsionally aligned, vertical disparities will be zero on the horizontal meridian^17,18^.

Given these clear differences in the distributions of horizontal and vertical disparity in different image locations, we would expect the way that disparity is encoded by the visual system to vary in a similar way.

In V1, the distributions of binocular cell properties tend to vary with retinal eccentricity. Increasing eccentricity tends to result in an increase in both the mean magnitude and range of preferred horizontal disparity^19,20^. Joshua and Bishop^20^ reported a slight decrease in the range of preferred vertical disparities with increasing eccentricity along the horizontal meridian, while Durand *et al*.^19^ reported an increase in vertical disparity distributions with increasing eccentricity when measurements were taken away from the horizontal retinal axis. These results are not contradictory, as vertical disparities are expected to be greater in both more eccentric and more vertically displaced (from the horizontal meridian) locations (see e.g. refs^17,18,21^). The range of tunings for horizontal disparities is greater than that for vertical disparities at all measured eccentricities^16,19^.

Similar effects have been observed in other areas of the visual cortex. In V4, disparity tuning becomes broader as eccentricity increases^22^. Not all authors reported effects of eccentricity on the distribution of disparities; Prince, Cumming, and Parker^23^ observed little effect of eccentricity on responses to stimulus frequency or disparity sensitivity for binocular simple cells in macaque V1, up to 4° of eccentricity.

The symmetry of the disparity response function has been used to classify binocular neurons as ‘near’, ‘far’, ‘tuned excitatory’ and ‘tuned inhibitory’, depending on the shape of the tuning function. Studies of V1 have found that more than 50% of binocular neurons are tuned to detect features close to zero disparity^19,23,24^. Both Poggio and Fischer and Prince *et al*. found a slight bias towards tuning for far disparities. Durand *et al*. observed a substantial effect of eccentricity on the ratio of cell types. As eccentricity increased the proportion of TE and TI cells decreased and the proportion of near and far types increased in both horizontal and vertical disparities, the bias towards the horoptor was stronger in the vertical direction than in the horizontal direction^19^.

One difficulty in interpreting these results is that neurons are not equally well sampled across the visual field. Sprague *et al*.^10^ collated data across a number of studies and found that the majority of cells sampled from V1 were in the lower visual field. They analysed disparity tuning in these studies separately for cells in the upper and lower halves of the visual field. They found that cells in the lower visual field were more likely to be tuned to crossed disparities, while cells in the upper visual field were more likely to be tuned to uncrossed disparities. This difference is consistent with the distributions of disparities that they measured in humans. In this study, we investigated the effects of image location on the encoding of binocular information using Independent Component Analysis^25^. ICA imposes a highly kurtotic prior on neural responses, with the goal of producing a sparse encoding of natural images^26^. When applied to patches taken from natural binocular images, it is known to produce sparse components with some similarities to simple-cell receptive fields in V1^27,28^.

We used ICA as a model of the first neural layer of encoding binocular images. This encoding, by itself, is unable to provide unambiguous tuning to binocular disparity. This is because the linear filters that it generates are sensitive to other properties of the image, such as its local phase. However, it has been proposed that layers in a neural network should alternate between selectivity and invariance to such features^29,30^, and this approach has been taken to creating multi-layer models of the cortical encoding of natural images^31^. In the case of binocular disparity, the binocular energy model^32^ creates disparity tuning that is invariant to local phase and position by combining the first layer (simple cell) filters to generate the second layer (complex cell) responses. This model is an idealisation of the actual response properties of cortical neurons. For example, it is possible to create phase invariance by combining multiple simple cells with a range of phase tunings, rather than requiring pairs of neurons with a quadrature phase relationship^33^. Also, there is evidence that complex cell responses obtain some input direct from geniculate neurons, rather than from simple cells, and that invariance can be achieved through intradendritic computations (for a review see ref.^34^), so this hierarchical model might best be seen as a idealisation. Nevertheless, we have shown that it is possible to learn such invariance in a two-layer network using Independent Subspace Analysis^35^. ISA extends ICA to learn complex-cell-like components from natural image patches by fixing an orthogonal *L*_2_ norm (the square root of the sum of squared responses) between the linear subunits of complex cells, while maximising response kurtosis across complex cell components^25^. Another approach to learning binocular neural responses has recently been proposed, focused on the specific goal of estimating binocular disparity^36,37^. In the current analysis, we concentrated on the use of ICA, to determine how spatial non-stationarity of the statistics of natural images might affect the initial encoding of binocular information.

Previous studies have investigated the effects of image location on binocular vision, and the optimal encoding of binocular information, from a number of different angles. Sprague *et al*.^10^ and Liu *et al*.^14^ measured actual depth. Burge and Geisler^36^ trained their model using samples with known depths. As the visual system has no *a priori* knowledge of depth in the scene, our study focused purely on the sorts of visual stimuli available in the retinal image, with no information about the corresponding depth. In machine learning terms, the training was unsupervised. The primary contribution of this paper is to demonstrate that the effects listed can be learned by an agent with no prior knowledge of the environment. It builds on our earlier work by considering the effects of image position in this learning.

Expansion of ICA to consider the effects of image position on the components learned is an important advance on earlier studies which have learned components without giving consideration to the image locations of the training samples used^27,35^. In computer vision, and in modelling biological systems, it is common to assume that image statistics are the same from one location to another. This greatly simplifies both learning and modelling, as a single set of receptive fields can be learned for the whole retina, rather than individually for each location^38^. However, this approach relies on the simplifying assumption that the statistics are the same at all locations and, especially in the case of binocular statistics and receptive field properties, as detailed above, we know that this is not the case.

To investigate the effects of spatial location on the components learned through ICA, we divided the images into regions defined by eccentricity and quadrant, and performed Independent Component Analysis^25^ on image patches sampled from these regions. We then analysed differences between the left and right parts of the components to determine how these components would respond to binocular disparities. This analysis allows us to determine how the first, linear, stage of binocular encoding varies with image location, and to link this variation to geometric predictions and to known properties of cortical neurons.

## Results

### Radial Eccentricity

#### Binocularity of components

Binocular components can exhibit a wide range of binocularity, ranging from completely monocular, where all the energy is contained within one of the component’s views, to completely binocular, where each view contains equal amounts of energy. This is measured using a binocular ratio between left and right component energies. The energy was defined as the sum of squares of the component weights (filter pixels) rather than the energy of the fitted Gabor functions; as a result, the ratios are unaffected by the outcome of fitting a Gabor function. We defined a binocular component as having a binocular ratio of greater than or equal to 0.25, meaning one component has no more than twice the energy of the other, and monocular components as all components with a binocular ratio of less than 0.25.

**Figure 1:**
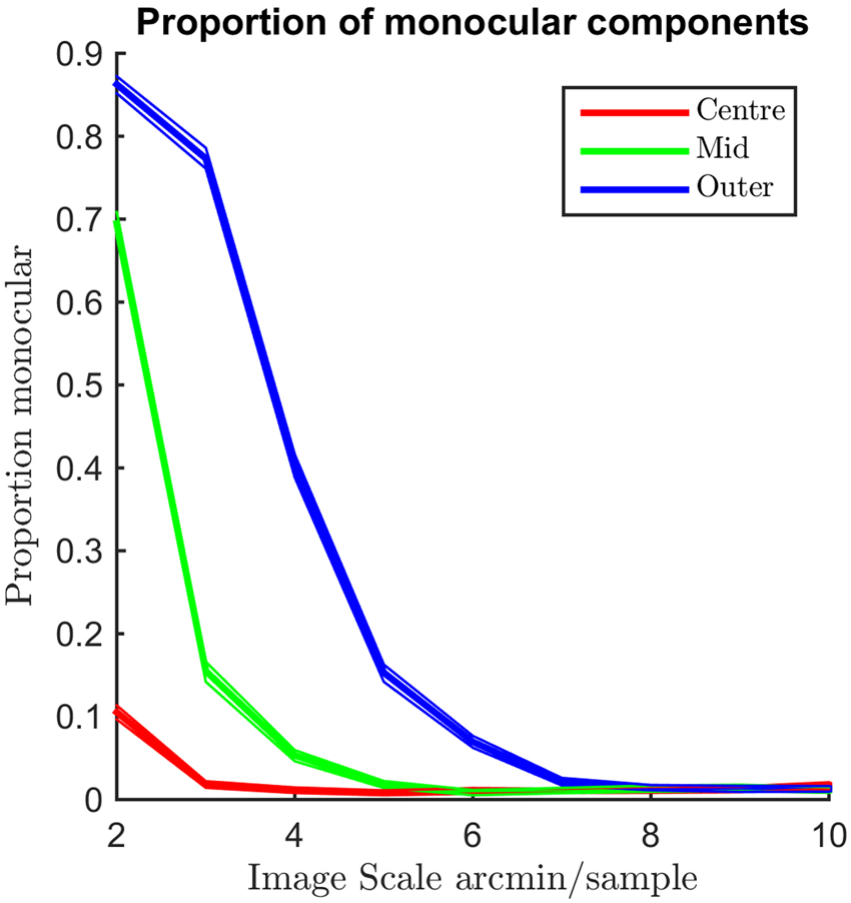
Bootstrapped histograms of the proportion of components with a binocular energy ratio of less than 0.25 across image scales, as a function of eccentricity and sample resolution. The median of the bootstrapped distributions is shown as a thick line, the 95% confidence intervals are shown as thin lines. There is a strong trend toward a larger proportion of binocular components at coarser sample resolutions. Similarly, at sample resolutions less than 8 arcmin/sample there is a clear increase in monocular components as eccentricity increases.

The ratio of energies between the left and right parts of the ICA components varies throughout the component sets. As shown in Fig. 1, the proportion of monocular components decreases as the sample grid becomes coarser, and increases with eccentricity.

At the finest resolution (2 arcmin/sample) the vast majority of components learned from the outer region are monocular. However, at the coarsest resolution measured, the vast majority of components are binocular at all eccentricities. The proportion of monocular components is lower for both ‘mid’ and ‘centre’ regions than for the ‘outer’ region at all resolutions below 8 arcmin per sample and the centre region has the lowest proportion of monocular components across all resolutions below 8 arcmin per sample. At resolutions of 8 arcmin per sample and above, the vast majority of components are binocular at all eccentricities and differences between eccentricities become insignificant.

In subsequent analysis we considered only components with both an energy ratio of greater than 0.25 and successfully fitted Gabor functions in both left and right parts of the components. The proportions of valid components varied according to both eccentricity and sample resolution. Both the lowest and highest proportion of valid components (66% & 84%) were found in eccentric regions at the finest scales, where the proportion of monocular components was highest. Otherwise ratios were consistently centred (median) around 77.9% (Median Absolute Deviations, 0.7%).

### Spatial Scale of Components

**Figure 2:**
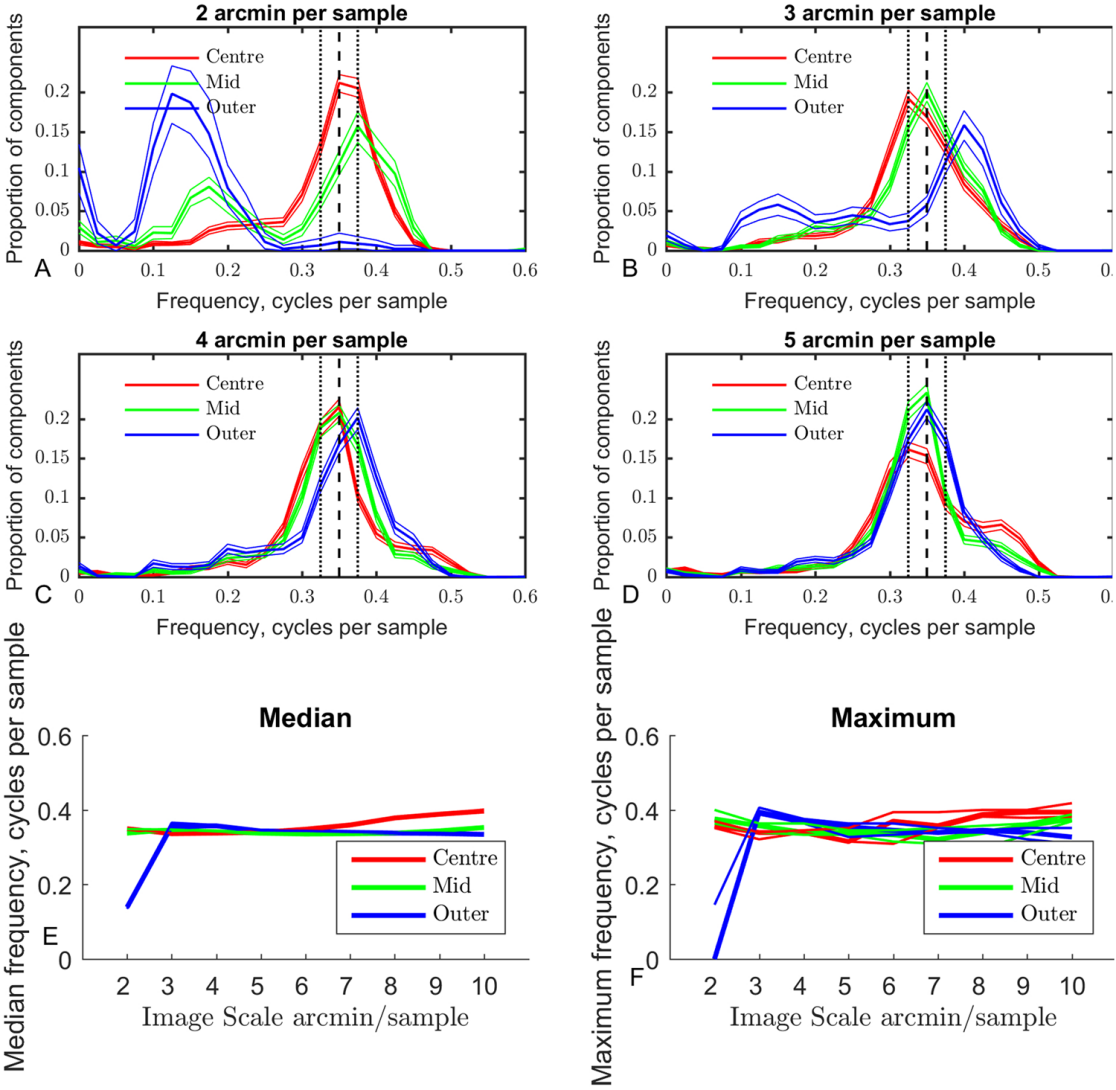
Distributions of component frequencies across eccentricity and sample resolution. (**A**–**D)** Show bootstrapped histograms of the distributions of component frequency across eccentricity at selected sample resolutions. The median of 200 bootstrapped distributions is shown as a thick line, the 95% confidence intervals are shown as thin lines. The finest sample resolution plot **(A)** shows the greatest difference between distributions across eccentricity. At the coarsest resolution examined, the distributions are highly similar across eccentricity. **(E)** Shows the median wavelength of the components across sample resolution and eccentricity. The medians of the bootstrapped distributions are shown as thick lines, the 95% CI as thin lines. The vertical axis shows frequency in cycles per sample, the frequency in cycles per arcmin can be found by dividing by the number of arcmin/sample. A clear trend can be seen in **(E)** as the median frequency in the central region becomes higher as the sample resolution becomes coarser. Similarly, the difference between medians across eccentricity becomes more pronounced as the sample resolution becomes coarser. The maximum frequency across eccentricity and sample resolution is shown in **(F)**. As with the median frequency, a significant difference can be seen at the finest scale in the outer category, otherwise no significant trends can be observed, maxima are generally within the 95% CI of each other.

The distributions of frequency across eccentricity and sample resolution are shown in Fig. 2. The distributions of frequency are shown for four selected scales in Fig. 2A–D. The frequencies are measured relative to the sampling grid in cycles per sample, values may be converted to cycles per arcmin by dividing by the sampling resolutions. At most resolutions, component frequencies are clustered around a similar frequency for all eccentricities. Figure 2E,F show the median and maximum of the frequency distributions across resolution for the three eccentricities. For most resolutions there are no substantial or significant differences between maximum frequencies across eccentricities. An exception is the 2 arcmin/sample resolution, where the peak of the frequency distribution for components in the outer region is substantially lower than for components learned from the mid and centre regions. Examination of Fig. 1 shows this region is dominated by monocular components indicating that binocular matching performance is poor. In these circumstances we would expect low frequency features to be more closely correlated than high frequency features, explaining the dip. It is worth noting that, as the graph plots the function in terms of sampling resolution, this frequency is relatively high at ≈0.3 cycles per arcmin (18 cycles/degree). The highly similar distributions of frequency across most resolutions and eccentricities suggests that the frequency distributions are a consequence of the band-pass filtering in the ICA analysis rather than a feature of natural images, as these tend to follow a 1/*f* ^*α*^ curve. The substantially lower maximum for the distribution of frequencies in the outer region at the finest sample resolution, combined with the high proportion of monocular components in this case, suggests that binocular matches within the sampling windows used are uncommon at these ranges, likely as a result of the geometry of binocular images creating relatively large disparities at more eccentric regions.

**Figure 3:**
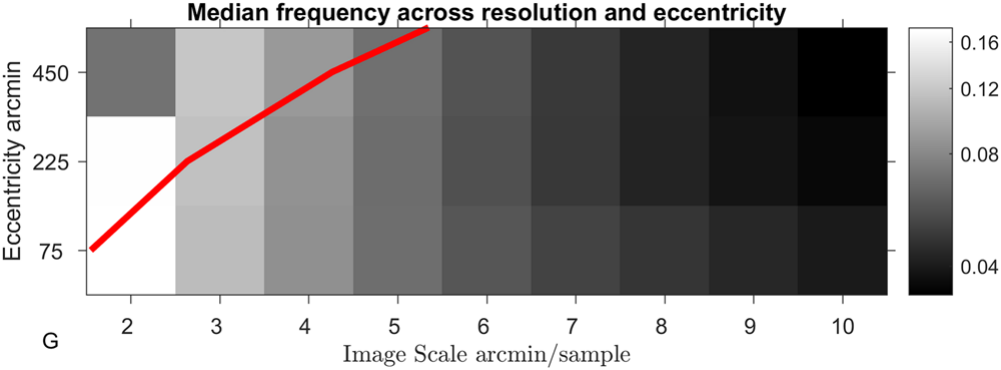
The median frequency across resolution and eccentricity replotted as a heatmap. The estimated resolution of midget retinal ganglion cells^40^ is shown as a red line on the plot. This line marks a boundary between stimuli detectable by mRGC (below the line) and stimuli than cannot be detected by mRGC (above the line).

We performed ICA analysis directly on binocular image patches in order to determine statistical properties of binocular natural stimuli while reducing the number of assumptions made about the human visual system. The imaging resolution available to the visual cortex is constrained by the physiology of the retina and subsequent connections between the retina and the visual cortex. Signals generated by photo-sensitive cells are processed by retinal ganglion cells (RGC) prior to transmission upstream towards the LGN and the visual cortex. The density and receptive field structure of retinal ganglion cells in individual retina imposes limits on subsequent processing when monocular signals are combined by binocular tuned neurons in the visual cortex^39^. We have plotted the estimated density function of midget retinal ganglion cells (mRGC) in relation to the sampling resolution of our technique at the chosen eccentricities in Fig. 3. Sample resolutions below this line are less dense than mRGC neurons in the retina, so we can be confident that human physiology is capable of detecting and utilising frequencies of components learned at these resolutions. Above this line the sampling resolution is denser than mRGC neurons so the algorithm is theoretically capable of learning components with frequencies higher than those transmitted to the visual cortex.

### Position Disparity

**Figure 4:**
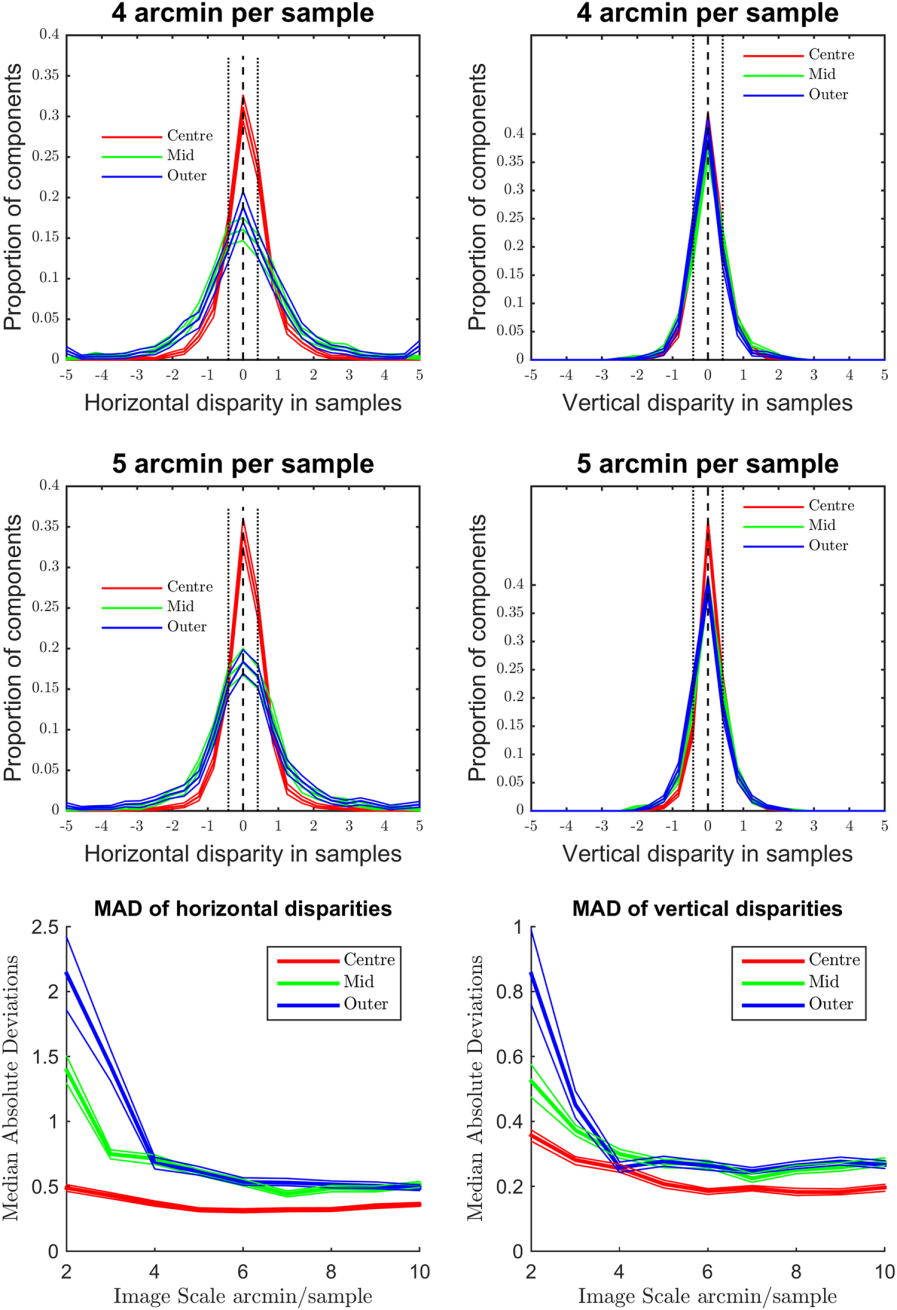
Distributions of horizontal and vertical position disparity across eccentricity. (**A**,**C)** Distributions of horizontal position disparity across eccentricity at 4 **(A)** and 5 **(C)** arcmin per sample. Thick lines show the median of bootstrapped histograms, thin lines show the 95% confidence intervals. (**B**,**D**) Distributions of vertical position disparity across eccentricity at 4 (**B**) and 5 (**D**) arcmin per sample. (**E**,**F**) Median of Absolute Deviations in horizontal (**E**) and vertical (**F**) position disparity across sample resolution at each eccentricity region.

Position disparity is measured in terms of the relative locations of the receptive fields (the windowing function of the Gabor) of the left and right components. Horizontal disparity measures the relative receptive field locations along the axis parallel to the interocular shift; vertical disparity measures the relative receptive field locations along the axis perpendicular to the interocular shift. As detailed in the methods section, position in our images is measured using a spherical, pin-hole camera coordinate system, such that the horizontal and vertical direction correspond to angular measures of azimuth and elevation, respectively. Individual components can exhibit a mix of both horizontal and vertical position disparities. The distributions of horizontal and vertical disparities at two sample resolutions (4 and 5 arcmin/sample) are shown in Fig. 4A–D. Distributions of both vertical and horizontal position disparities follow double exponential distributions with 0 mean. Significant differences in the peak and spread of these distributions across eccentricity can be seen, especially for horizontal disparities (Fig. 4A,C). Plots of the median of absolute deviations (MAD) are shown for horizontal (Fig. 4E) and vertical (Fig. 4F) disparities. These plots show how the spread of the distribution changes over sample resolution. Distributions of horizontal disparity are more peaked at coarser (larger) sample resolutions, they are also progressively more peaked in the centre and middle sections of the image than in the outer region. Vertical disparities follow a highly similar pattern to horizontal disparities, but the MAD of vertical distributions is generally about half the MAD of horizontal distributions, suggesting that the vertical disparities tend to be substantially smaller than horizontal disparities, as expected.

### Phase disparity

**Figure 5:**
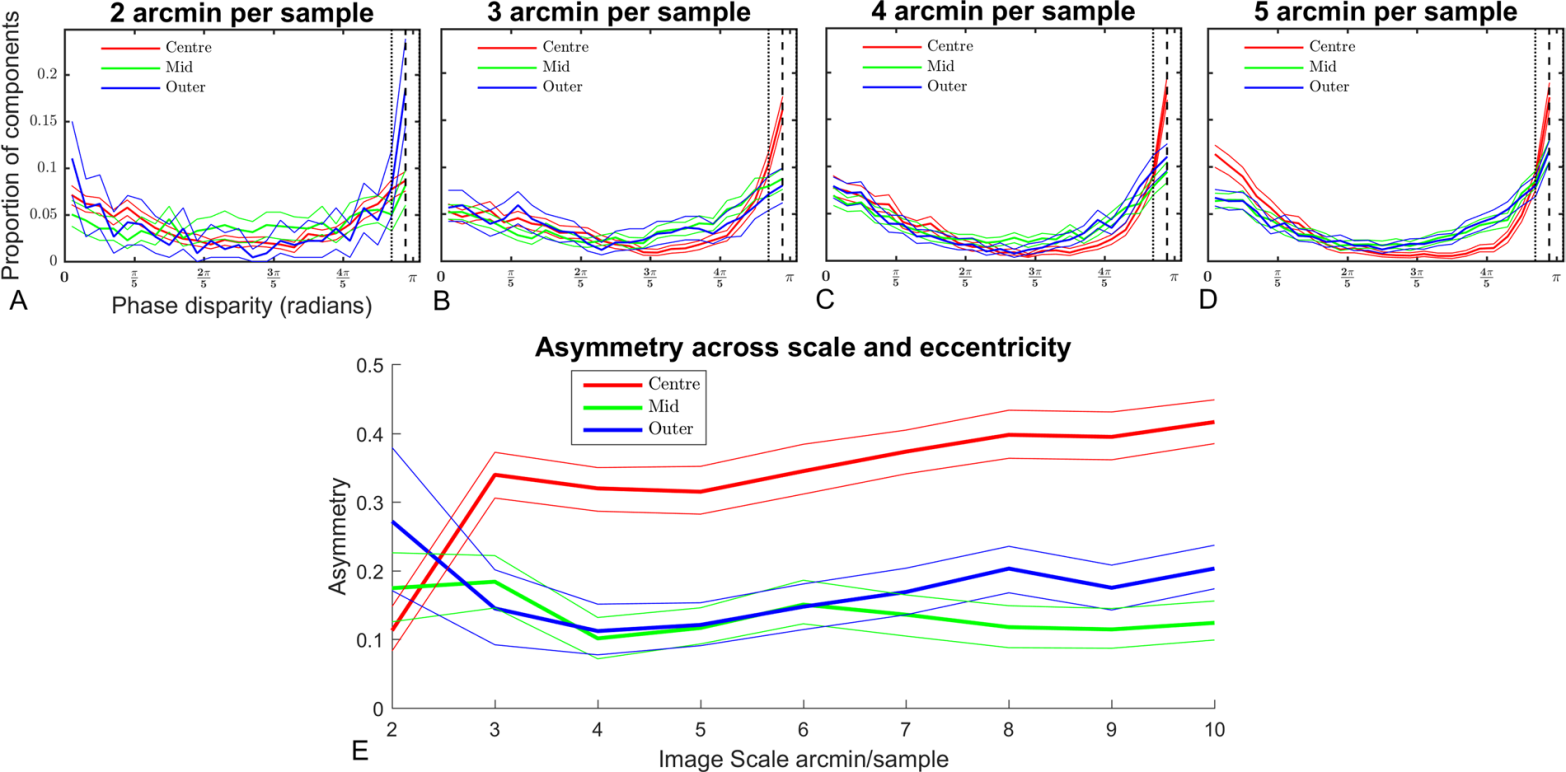
Phase disparity across eccentricity and sample resolution. (**A**–**D)** Bootstrapped histograms of phase disparity, thick lines show median of 200 distributions, thin lines show 95% confidence intervals. Phase disparity distributions are shown at four scales, the finest scale 2 arcmin per sample **(A)**, 3 **(B)**, 4 **(C)** and 5 arcmin per sample. Plot **(E)** shows the asymmetry across sample resolutions of the phase disparity distributions for the three regions. A clear effect of eccentricity can be seen at most sample resolutions where the distribution of phase disparity is more asymmetric in the centre region that at more eccentric regions **(E)**. Subplots **(B**–**D)** show that the distributions of phase disparity are biased towards *π* radians phase disparity.

Distributions of absolute phase disparity across eccentricity for selected scales are shown in Fig. 5A–D. Across all measured phase distributions there are clear peaks at 0 and *π* phase, however these peaks are highly asymmetric. Across many scales and eccentricities the largest peak in the distributions is at *π* radians phase disparity, indicating components tuned to detect anti-correlated features in each view.

To quantify the size of the difference in the peaks at 0 and *π*, we calculated the asymmetry of each distribution. Figure 5E shows the changes in the asymmetry of the phase distributions across eccentricity and scale. Asymmetry is measured as the absolute difference between a distribution and its mirror around a central point, in this case *π*/2. For a normalised distribution, the maximum sum of absolute differences is 2.0 (a maximum of 1, plus its mirror image), so the absolute sum is divided by 2. With this metric, a perfectly symmetric distribution will have an asymmetry of 0, a function with all its energy in one half of the distribution will have an asymmetry of 1.

At sampling resolutions of 3 arcmin/sample and greater, a strong difference in asymmetry is apparent between components learned from central regions and components learned from more eccentric regions. The phase disparity of components learned from central regions is much more strongly asymmetric than in Mid or Outer regions and there is a slight increase in asymmetry with decreasing sample resolution. However at the finest sampling resolution this is reversed, with the centre region having the smallest phase asymmetry.

The asymmetry measure is only sensitive to the magnitude of asymmetry rather than the nature of the asymmetry. Examination of Fig. 5A–D shows that where asymmetries occur they are generally biased toward *π* radians, that is anti-correlated components. This includes the 2 arcmin/sample resolution where the effect of eccentricity is reversed. Overall, Fig. 5 indicates a strong effect of eccentricity on the symmetry of phase distributions and a clear bias toward anti-correlated components in the central regions of the binocular images. This effect becomes more pronounced as the sample resolution decreases and the coarseness of features increases.

### Orientation disparity

**Figure 6:**
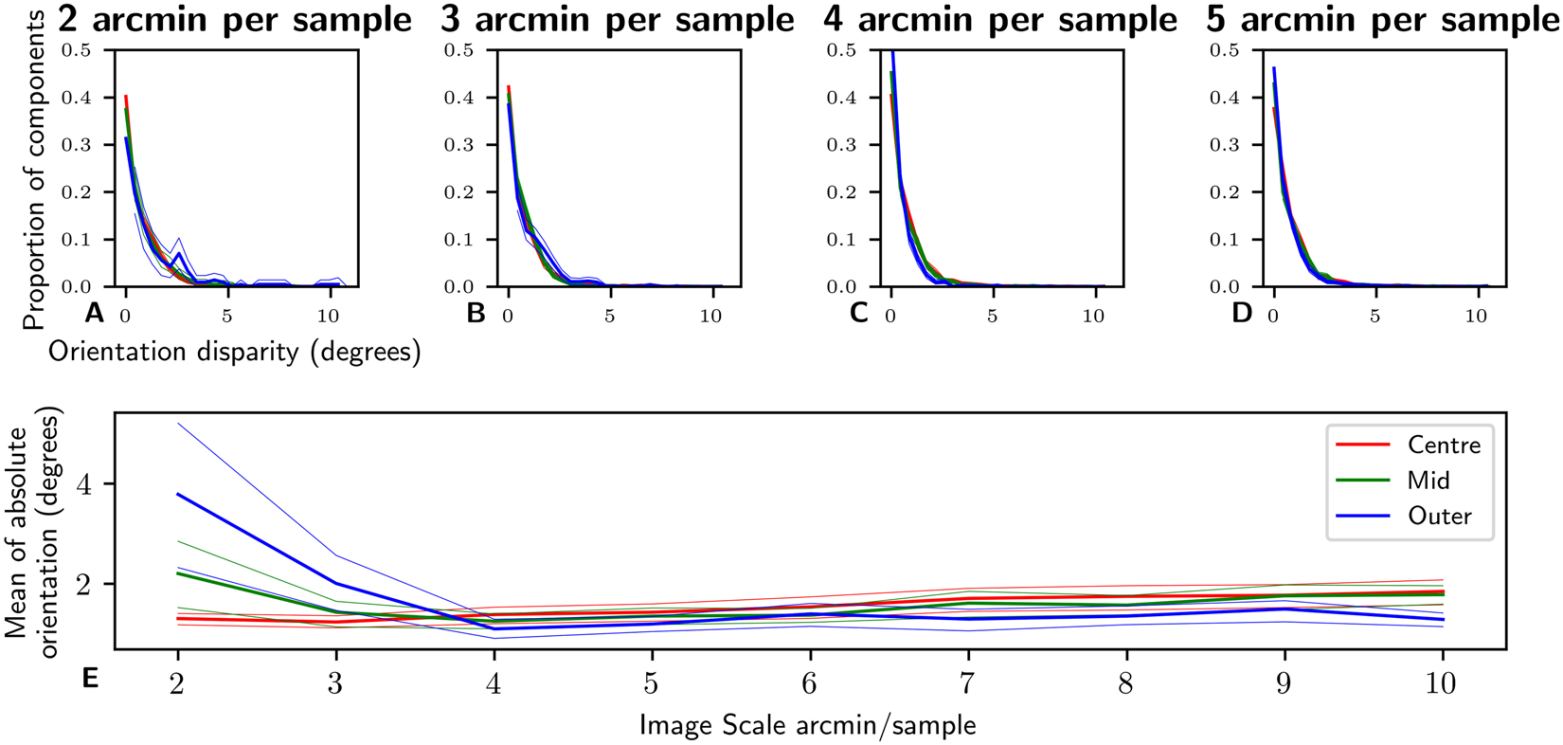
Distributions of orientation disparity across resolution and eccentricity. Subplots **(A**–**D**) show the distributions of orientation disparity for 2 to 5 arcmin/sample resolutions. The distributions are strongly biased towards zero orientation disparity, with almost all components exhibiting an orientation disparity of less than 5°. In general there is little effect of eccentricity on the distributions of orientation disparity across all scales. Subplot (**E**) shows the MAD of the absolute orientation distributions. For more coarse scales (>4 arcmin/sample) there is little to no significant effect of eccentricity on the distributions of orientations. However a small effect does emerge at finer scales with the distribution of orientations broadening at larger distributions, the effect is small <3°.

Orientation disparity is calculated as the absolute difference in orientation between left and right fitted Gabor functions. The distributions of orientation disparity are plotted in Fig. 6. The distributions across sample resolutions between 2 and 5 arcmin/sample are shown in Fig. 6A–D. The distributions follow a pattern similar to an exponential distribution, with a very strong bias towards 0. Across all 4 resolutions, no significant differences between distributions are visible across eccentricity. Figure 6E shows the MAD of the orientation disparities across resolution and eccentricity. At sample resolutions coarser than 4 arcmin/sample there is little change in MAD across either resolution or eccentricity, although there is a small but significant difference between Outer and Centre regions at many resolutions (4, 7, 8, 10). At fine sampling resolutions the spread of the orientation disparities in the Outer region is statistically larger than for the Centre region, however visual inspection of the distributions (Fig. 6A) shows this to be a very small difference. This may partially be explained by the fact that the proportion of binocular components is much lower for 2 arcmin/sample resolutions in eccentric regions than for other areas.

Although a small difference between orientation disparity distributions across eccentricity was detected at the finest sample resolutions, little effect of either sample resolution or eccentricity was found at sample resolutions of 4 arcmin/sample or greater. Across all sample resolutions and eccentricities, orientation disparity was strongly biased towards zero, all distributions had a MAD of less than 4.6 degrees.

### Eccentricity and Quadrant

**Figure 7:**
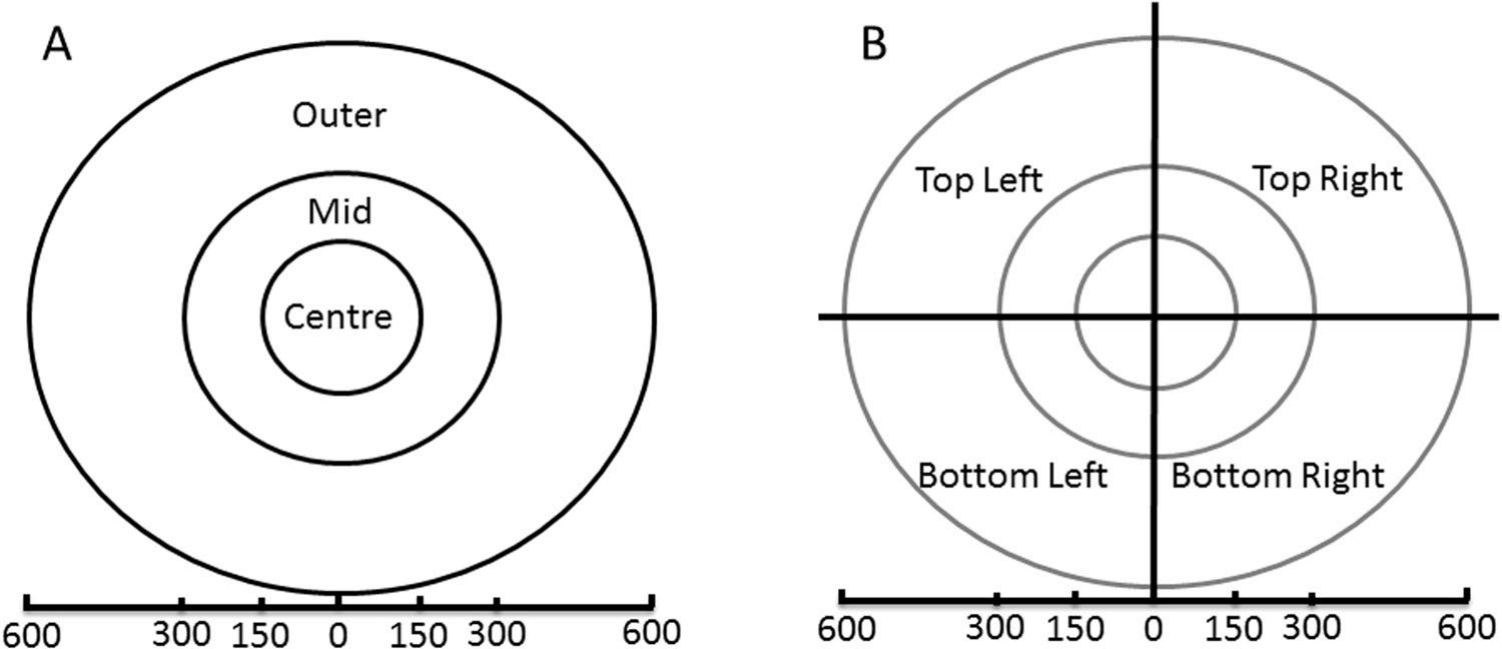
(**A**) The regions used in the first analysis, defined according to the distance from the centre of the image. **(B)** These regions were combined with quadrant partitions in the second analysis. The four quadrants combine with the three eccentricities to form 12 regions in total.

The geometry of vergence predicts different signs of vertical disparity depending on retinoptic location (see e.g. refs^17,18,21^). In particular, we would expect different distributions depending on the quadrant from which a distribution was sampled. These predictions have been confirmed for natural binocular images for active human observers^10^. These results also showed a clear difference in the distribution of horizontal disparity between the upper and lower halves of the visual field. To assess the effect of these predicted asymmetries on the binocular properties of independent components, we defined four quadrants (top left, top right, bottom left and bottom right) by dissecting the binocular image pairs horizontally and vertical through the focal point in the centre of the images. As we would expect the effects of vergence to also be dependent on eccentricity we further sub-divided the images into the three eccentricity regions used earlier. The four quadrants and the three regions of eccentricity permute to 12 separate regions as shown in Fig. 7B. We selected a single representative sample resolution of 4 arcmin/sample for the analysis.

#### Monocular components

**Figure 8:**
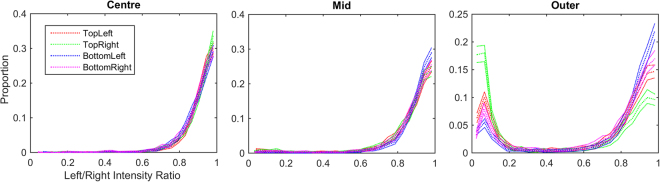
Distributions of left/right intensity ratio for all fitted Gabor functions in each region. The three plots divide the distributions by eccentricity. Within each plot the distributions of left/right intensity are shown for samples drawn from the intersection between each quadrant and the eccentricity of the plot.

As with the previous eccentricity analysis, a proportion of components in each region will be monocular at this sample resolution. The ratio of intensity between left and right components for all successfully fitted Gabor functions within each region is shown in Fig. 8. Components with an intensity ratio of less than 0.5 are considered monocular, components with a ratio greater than or equal to 0.5 are considered binocular. All regions produce significant numbers of binocular components that can be used in the subsequent analysis. At smaller eccentricities, almost no monocular components are produced in any quadrant. Only in the most eccentric regions do substantial numbers of monocular components appear, however large quantities of binocular components are also produced. For the two inner regions, little difference is discernible between the distributions of intensity ratio across quadrants. In contrast, the ‘outer’ region shows a clear effect of quadrant, such that the proportion of binocular components is significantly higher in the bottom quadrants than in the upper quadrants. A left/right effect is also visible, with quadrants on the right of the centroid producing more monocular components than quadrants on the left.

#### Spatial Scale of Components

**Figure 9:**
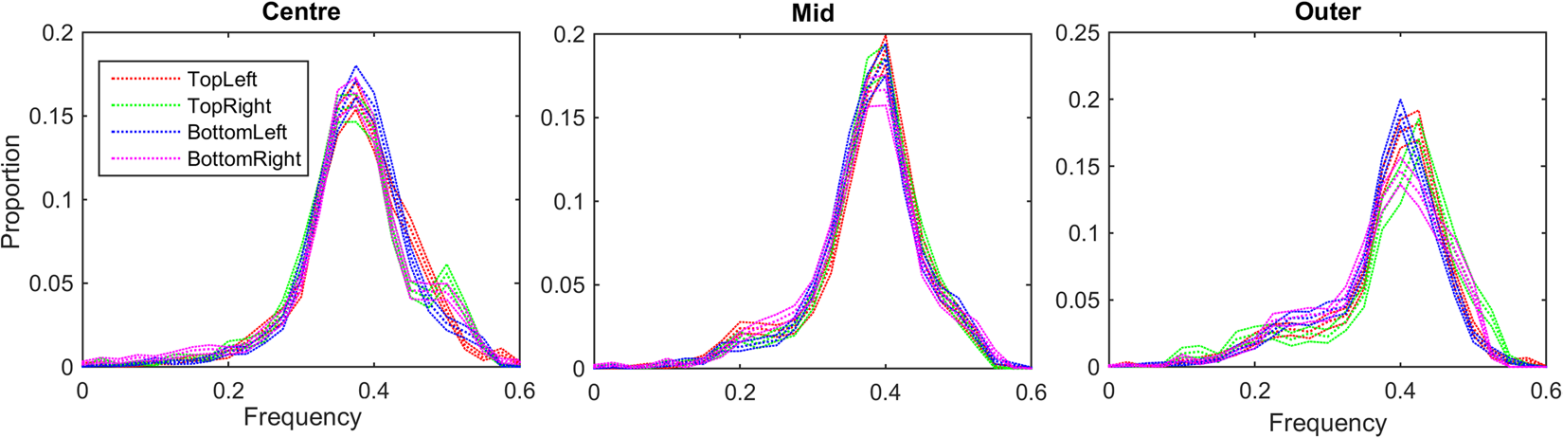
Distributions of component frequency across quadrant for the three eccentric regions. Distributions are all highly similar across quadrant for each eccentricity.

Although there is an effect of eccentricity on the distribution of component frequencies (see Fig. 2) there is no visible effect of quadrant on the distributions of frequency (Fig. 9). The distributions of frequency show no significant change across quadrant.

#### Position disparity

**Figure 10:**
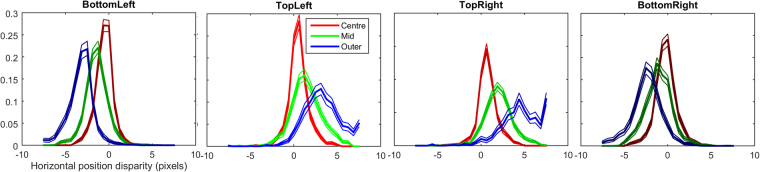
Distributions of horizontal position disparity across eccentricity for each of the four quadrants. The legend for all four plots in found in the top left plot. Clear effects of both eccentricity and quadrant are visible. For the top left and top right quadrant plots there is a clear progression towards positive horizontal position disparities with increasing eccentricity. For bottom left and bottom right quadrant plots a progression can be seen toward increasingly negative horizontal position disparities with increasing eccentricity.

The distributions of horizontal position disparity for each quadrant and eccentricity are shown in Fig. 10. The plots show a clear difference in the distributions between quadrants above the centroid and quadrants below the centroid. For the top-left and top-right quadrants the distributions become increasingly biased towards far/uncrossed disparities (positive values). The bottom-left and bottom-right quadrants exhibit the opposite trend, with an increase in near/crossed disparities (negative values) with eccentricity. There is no substantial difference between the respective left and right quadrants. This difference in the distribution of disparity tunings in the upper and lower visual fields reflects the disparity statistics reported by Sprague *et al*.^10^, and their analysis of the disparity tuning of V1 neurons.

**Figure 11:**
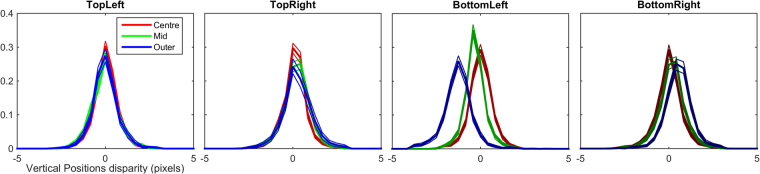
Distributions of vertical position disparities across eccentricity for each quadrant. For both top-left and top-right quadrants there is only a small and statistically insignificant difference between the distributions across eccentricity. For the bottom-left quadrant the vertical position disparity becomes increasingly negative with eccentricity. For the bottom-right quadrant the vertical position disparity becomes increasingly positive with eccentricity.

Distributions for vertical position disparity are shown in Fig. 11. For the quadrants above the centroid, little or no effect of eccentricity is apparent. For the quadrants below the centroid, a strong effect of eccentricity is visible, as is an effect of quadrant. For the bottom-left quadrant the distributions of vertical position disparity become increasingly biased towards negative disparities. For the bottom-right quadrant a similar effect is visible, however the direction of the effect is reversed, distributions of vertical position disparity become increasingly biased towards positive values. Negative values of vertical disparity indicate points that are lower in the left eye; positive values indicated the opposite. Again, these distributions reflect the pattern of vertical disparities recorded by Sprague *et al*.^10^.

It was previously observed that there is no relationship between horizontal and vertical component disparities across binocular images as a whole^28^. When partitioned by eccentricity and region this effect was preserved, Spearman’s rank correlation coefficient was not significant (p > 0.05) on all quadrants and at all eccentricities.

### Summary of key findings

Effects of radial eccentricity:

- At all but the coarsest resolutions (<7 arcmin/sample) eccentric regions consistently produce more monocular components than foveal regions.
- Across all measured resolutions horizontal and vertical position disparity tunings are more spread in eccentric regions than foveal regions.
- Across all resolutions variation in horizontal spread is more pronounced than variation in vertical spread.
- Across all but the finest resolutions (>3 arcmin/sample) distributions of phase disparity are substantially more asymmetric in foveal regions than eccentric regions.
- Across coarser resolutions (>4 arcmin/sample) distributions of orientation disparity are not affected by eccentricity.
  Effects of eccentricity and quadrant:
- At the greatest eccentricity, regions below the centre produce more binocular components than regions above the centre.
- There was no effect of quadrant on the distribution of the spatial frequencies of components.
- There was a strong effect of vertical location on the distribution of horizontal position disparities. Regions above the centre show a strong bias toward far-tuned (uncrossed) components. Regions below the centre show a strong bias toward near-tuned (crossed) components. The effect is identical on both sides of the centroid. This bias increases with increasing eccentricity.
- There was a strong effect of location on the distribution of vertical position disparities below the centre. For locations to the bottom left, vertical disparities are biased toward negative values; for locations in the bottom right, disparities are biased toward positive values.

## Discussion

We are interested in the impact of image location on the distributions of independent components of binocular natural images, in contrast to the typical approach of ignoring location, under the assumption that image statistics do not vary from one location to another^38^. Most of the effects that we found have simple and logical explanations given the geometry of verged binocular images, and have also been found in the disparities recorded for observers performing everyday tasks^10^. We have demonstrated that the variations in the statistics of binocular images affect the encoding of images through the unsupervised learning of independent components.

We would expect to find a greater number of zero or near-zero disparity tunings close to the point of vergence, compared with eccentric regions. Similarly, we would expect to find a greater proportion of monocular components at eccentric regions compared to central regions. This is what we observe (Fig. 2) at fine resolutions. For the finest resolutions, almost no binocular components are found in the most eccentric region, at coarser resolutions the vast majority of components are binocular even in eccentric regions. This is consistent with the idea that disparities are generally larger at eccentric regions than central regions, as the larger disparities are detected and coded in the coarse resolution components, but the high frequency disparities coded by the fine resolution components are generally rarer in eccentric regions compared to central regions.

Similarly, a greater range of distance variation further away from the object of focus would be expected to produce a greater spread of horizontal position disparity with increasing eccentricity (see Fig. 4). The scale of the position disparities would be expected to decrease with decreasing sample resolution as the disparities are rescaled along with the image. The distributions of actual scene disparities would not be expected to be uniform across the image, the ground-plane and objects placed upon it would be expected to be closer to the viewer than objects above this plane. Ground-plane effects are likely to explain the difference in distributions of horizontal potions disparities above and below the image centroid (Fig. 10).

The expectations for vertical position disparities are different than for horizontal position disparities. The distributions of vertical disparities in natural images vary across image quadrants^10,17^. Positive disparities are expected in the top left and bottom right quadrant, negative disparities are expected in the other two quadrants. We found the predicted effects of location on the vertical disparities of components in the lower visual field, but no differences in the upper visual field.

Although focus, vergence and scene structure can account for the variations in proportions of monocular components, distributions of frequency and position disparities, they do not account for variations in phase symmetry. As the underlying distributions of phase in each individual view are not affected by the focus or vergence we would expect the distributions of phase disparity to be affected by local disparities alone. The bias towards anti-correlated components, especially in the central region where highly correlated, low disparity components would be expected to predominate, is unexpected. Previous research has found a bias towards anti-correlated components in ICA analysis of binocular images^28^ when samples are taken from across the entire image. The distributions of physical disparities will be scaled along with the images when the sample resolution is decreased. The proportion of anti-correlated components was not strongly affected by the sample resolution of the image, indicating that the proportion of anti-correlated components is not directly related to the underlying distribution of disparities.

A number of roles for anti-correlated components have been suggested; Burge and Geisler^36^ found that anti-correlated components in their model of binocular disparity coding could signal the presence of a disparity by not responding. Read and Cumming^41^ suggested an inhibitory role, where anti-correlated components veto ‘impossible’ disparities. As ICA is an efficient coding method rather than an encoding optimised specifically for estimating binocular disparity (such as the algorithm of Burge and Geisler^36^) it is agnostic to the true scene disparity. An ICA component can respond to false as well as true matches in the binocular images. If we consider the ideas of Read and Cumming^41^, it is possible that ICA is producing more false matches in the centre of the image where disparities are small, than in the periphery.

Binocular complex cells in V1 are typically modelled as generating their responses from multiple simple cell inputs^42^. Unlike simple cells, complex cells are tuned to detect a particular disparity regardless of local position (phase). A naïve model could construct a binocular complex cell from a set of simple cells tuned to detect similar features at slightly different positions spanning the receptive field. This model would correctly respond to stimuli with the desired disparities, however it would also respond to many other stimuli. Anti-correlated simple cells are required to suppress these false responses^16,42^. Larger numbers of anti-correlated simple cells would lead to greater inhibition of false-matches and finer tuning of complex-cells. This could result in some improvement in acuity in the foveal region of vision as the proportion of anti-correlated cells is higher than in the periphery. However Prince *et al*. found the proportion of anti-correlated cells in the striate cortex to be lower than the proportion of correlated cells^23^. Howarth *et al*. found inhibitory interactions between left and right eye view in the LGN of mice^43^, it is possible that lateral inhibition prior to V1 accounts for the lack of anti-correlated cells while still allowing for anti-correlation to play a role in the formation of complex-cell responses.

In studies which have sought to optimize binocular encoding specifically for the purpose of classifying the sign of binocular depth^36,37^ tuned-inhibitory units have been argued to play a potentially useful role in the depth discrimination. The lack of response from such a neuron can provide clear evidence for the presence of a signal with a particular disparity. In a similar vein, Read and Cumming^16^ argued that strong responses from these units provide evidence against the presence of signal with the disparity to which they are tuned. Goncalves and Welchman^37^ found a greater proportion of tuned-inhibitory type units when their network was trained on the task of disparity discrimination, than when the training simply seeks to encode the information present in binocular images, as found here and in our previous studies^28,35^. This however contrasts with the findings from cortical recordings, in which tuned-inhibitory neurons are rare^23^. These differences reflect the fact that the encoding of binocular information, through ICA and the brain, involves more than just the discrimination of disparity.

The distributions found in this study show some significant similarities with physiological measurements. In common with physiology, the preferred horizontal disparity increases with eccentricity^19,20^, and is biased towards crossed disparities in the lower visual field and uncrossed disparities in the upper visual field^10^. Similarly an increase in preferred vertical disparity has been found in the macaque^19^. Our analysis found a wider spread of horizontal disparities than vertical disparities matching physiological observations^16,19^. However, in common with previous studies^28,44^ significant deviations from physiology are observed. Studies of the phase tuning of binocular cells have found little evidence for substantial numbers of anti-correlated binocular cells in the visual cortex^23^, in contrast to the prevalence and even bias towards such components in the ICA analysis for central areas of the binocular image pairs. Although our analysis of the ratio of binocular energies in the components shows a strong effect of both scale and eccentricity, physiological measures have not found any effect of eccentricity on ocular dominance^45^.

This study has been concerned with the statistical properties of visual stimuli as they are presented to the visual system. In particular we are interested in the interaction between the binocular verged configuration of human vision and its impact on peripheral stimuli. It is important to note that the retina does not uniformly sample visual stimuli across its surface. Peripheral stimuli are under-sampled on the retina compared to fovea stimuli^46^. Peripheral acuity is further reduced by lower density of retinal ganglion cells compared to the fovea^40^. The minimum wavelength detected by retinal ganglion cells is generally lower than the wavelengths of the features analyzed. Using the calculations of Watson (2014)^40^ we estimated the minimum detectable wavelengths to range from ≈4.3 at an eccentricity of 10° to ≈0.009″ at the fovea (although calculations at the fovea are unreliable due to measurement difficulties, see ref.^7^). These ranges are below the sampling rate in all but the finest scales (<4 arcmin per sample) at the largest eccentricities.

The methods described here suffer from a number of limitations. Not all of the components admitted valid Gabor functions to be fitted in both parts of the components, resulting in the rejection of a proportion of components, although this proportion was low (median 23%). While this limits the study to examining only components that can be described using binocular pairs of Gabors, this is also common is physiology where single cell recordings are accepted for analysis where they respond both to binocular stimuli and in a manner that fits the assumptions of the analysis. Prince *et al*. for example analysed only successful Gabor fits^23,47^. ICA does not consider either internal or external sources of noise^48,49^, in this analysis the impact of high frequency noise was reduced using PCA whitening^25^. ICA attempts to form an efficient representation of the image such that it can be reconstructed, ICA does not consider how this representation will subsequently be used.

### Conclusions and further remarks

We have used independent component analysis to produce an energy efficient coding of the binocular visual scene for regions demarcated by eccentricity and location relative to the centre. The distributions of the components produced by this analysis were analysed in terms of their degree of binocularity, their frequencies, horizontal and vertical position disparities and their phase disparities.

We found that the distributions showed close matches with our expectations based on scene geometry, known distributions of neurons from physiological recordings and previous statistical analysis of eccentricity in binocular natural images.

Our results show substantial effects of both eccentricity and location on the distributions of ICA components in natural images. The theory that the brain is attempting to form an efficient coding of visual stimuli (Barlow, 1961) would suggest that distributions of receptive fields in the visual cortex would be similar to the distributions observed using an efficient coding mechanism such as ICA.

In common with previous similar analysis^28,44^ we found substantial similarities between the distributions of ICA components and physiological measurements, in particular the relationship between horizontal and vertical position disparities and measurements of preferred disparity in the visual cortex^10,19^. However, in common with previous research we also found substantial differences, in particular with respect to the distributions of phase disparity and proportions on monocular components. It is also important to note a number of other differences that have been outlined in other research, such as substantially narrower frequency and orientation bandwidth tuning in ICA than observed in the visual cortex^44^. That significantly larger numbers of anti-correlated components have been found in ICA than in physiological measures has previously been observed by Hunter and Hibbard^28^.

Binocular acuity in humans degrades rapidly with eccentricity^8,50^. The rate of decline in stereo-acuity is greater than that of monocular resolution^8,51,52^, however it is within the range of other monocular measures such as grating acuity^53^, and Vernier acuity^53,54^. The role that the underlying statistics of binocular images play in this overall decline is not yet fully understood. In this paper we have argued that variations in position disparity tuning in the visual cortex can be explained using the statistics of natural images. Variance in other binocular attributes, such as stereo-acuity, have not been tackled by this study and remain for future research.

## Methods

### Data-set

To investigate the effects of eccentricity on visual images our image set needs to simulate the setup of human binocular vision: the two views of the scene need to be separated by an appropriate interocular distance, and the two cameras need to be converged and focused on a suitable point. We used the data-set of Hibbard^55^ consisting of 139 pairs of images of scenes containing varying ranges of depth and object complexity. A horizontal camera mount holding two Nikon Coolpix 4500 digital cameras allowed manipulation of both the inter-camera separation and the cameras’ orientations around the vertical axis. For each captured scene, the cameras were oriented and focused on an object situated roughly in the centre of the images, this approximated binocular fixation on an object. Convergence of the human eyes affects the spatial distribution of both horizontal and vertical disparities^17,18,21^. Crucially the orientation of the cameras about the vertical axis induces vertical disparities that would otherwise be absent from an exclusively horizontally displaced camera setup. In all scenes the cameras were separated by 65 mm. The images were taken with symmetrically converged cameras, with zero elevation, and no cyclorotation. This is consistent with the fact that the expected cyclovergence under these conditions is negligible^56^. This does however mean that the geometry explored in the current analysis does not include the full range of distributions of horizontal and vertical disparities that would be experienced by an observer with fully mobile eyes.

Scenes captured in the binocular image set varied in both composition and range of disparities contained. As the distance from the cameras to the focal point varied due to scene composition the convergence of the cameras varied between image pairs; this in turn impacts upon the distributions of vertical disparities as these are caused by interaction of physical structure in the scene and the orientation of the cameras. A number of indoor scenes consisting of a mix of natural everyday objects, such as fruit and vegetables, were captured in a light-cabinet. A range of outdoor scenes were captured in the area in and around the town of St Andrews in Scotland, these included a range of beach and woodland scenes. The full set of images collected can be downloaded from www.github.com/DavidWilliamHunter/Bivis.

The captured images were calibrated to account for the lens and colour characteristics of the camera. We used the Camera Calibration Toolbox for Matlab^57^ to calibrate for the optics of the cameras’ lenses, and transformed the images such that they approximated an image taken with a perfect ‘pinhole-camera’. A consequence of this transformation is that we can describe pixels in terms of the visual angle they subtend, and position is defined in terms of this spherical coordinate system. The images were captured at a resolution of 1600 × 1200 pixels prior to calibration and reduced and calibrated to 1201 × 1201 pixels, where each pixel covered 1 arc minute of visual angle. The images were converted to CIE LAB values^58^ using colour patches captured from a Macbeth Colorchecker DC chart to establish the colour characteristics of the camera.

### Spatial Sampling

In the first analysis, we investigated the effects of eccentricity by dividing the images into three doughnut-shaped regions according to the distance from the focus point. The three regions ranged from 0 to 150, 150 to 300 and 300 to 600 pixels from the centre of the image. We labelled these the ‘centre’, ‘mid’ and ‘outer’ regions, respectively (see Fig. 7A). Separate patch sets were cut from each image region, each patch set was whitened and ICA performed as described above to produce an ICA model for each region. The ICA models for each region were analysed separately to provide a comparison between each region of eccentricity.

In the second analysis, we investigated the combined effects of radial eccentricity and quadrant, as shown in Fig. 7B. We divided each of the centre, mid and outer regions into three quadrants, creating 12 separation image regions.

### Pre-processing

As we are primarily interested in the local structure of the image rather than local luminance or contrast, we centred and normalised the luminance ranges for the patches in each eye.

1 $${{\bf{x}}}_{l}=\frac{{\dot{{\bf{x}}}}_{l,i}-\langle {\dot{{\bf{x}}}}_{l,i}\rangle }{|{\dot{{\bf{x}}}}_{l,i}|},{{\bf{x}}}_{r}=\frac{{\dot{{\bf{x}}}}_{r,i}-\langle {\dot{{\bf{x}}}}_{r,i}\rangle }{|{\dot{{\bf{x}}}}_{r,i}|}$$

2 $$x=\frac{[{{\bf{x}}}_{l},{{\bf{x}}}_{r}]}{|[{{\bf{x}}}_{l},{{\bf{x}}}_{r}]|}$$

where ${\dot{{\bf{x}}}}_{l,i}$ is the *i* ^*th*^ vectorised patch taken from the left image of the binocular image pair. $\langle {\dot{{\bf{x}}}}_{l,i}\rangle$ is the mean of all pixels in the vectorised sample patch ${\dot{{\bf{x}}}}_{l,i}$ and $|{\dot{{\bf{x}}}}_{l,i}|$ is the magnitude of the *i* ^*th*^ image patch.

Binocular samples are constructed by concatenating the centred and normalised left and right patches. Here [**x**_*l*_, **x**_*r*_] denotes concatenation of left and right patch vectors.

After concatenation and normalisation the patches are whitened using Principal Component Analysis. In natural images the power spectrum tends to follow a 1/*f* ^*α*^ curve. Whitening adjusts the individual image patches such that the overall power spectrum is approximately flat. This increases the prominence of mid-range frequencies in the analysis, and reduces its domination by low frequencies. However, as higher frequencies are often dominated by noise, enhancing the power of these frequencies would also enhance the noise^59^. In order to increase the signal to noise ratio, these high frequencies were removed by truncating the PCA to 250 components. This truncation retained ≈87% of variance in each patch set.

### Sample Resolution

**Figure 12:**
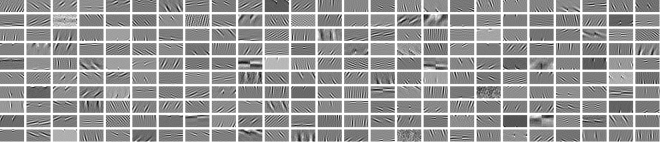
Example binocular ICA components. The components consist of two square patches, one for the left eye, one for the right eye, which are joined into a single rectangular component. Each component is surrounded by a white border. The components are scaled such that black represents the most strongly negative values and white the most strongly positive values; gray represents a value of zero. In turn impacts upon the distributions of vertical disparities as these are caused by interaction of physical structure in the scene and the orientation of the cameras. A number of indoor scenes consisting of a mix of natural everyday objects, such as fruit and vegetables, were captured in a light-cabinet. A range of outdoor scenes were captured in the area in and around the town of St Andrews in Scotland, these included a range of beach and woodland scenes. The full set of images collected can be downloaded from www.github.com/DavidWilliamHunter/Bivis.

The range of frequencies and therefore the scale of features that can be detected using ICA on image patches is constrained by both the ICA method, in particular the bandpass filtering in the whitening state, and by the size and resolution of the sample grid and the ICA method, in particular the bandpass filtering in the whitening stage^28^. The size of the patch in image space sets a lower bound on the frequencies that can be described accurately by a component, similarly the sampling resolution in image space sets an upper limit on the frequencies (the Shannon sampling limit). We assessed the effects of scale on the distributions of components by resizing the images with a bicubic interpolation function using the MATLAB imresize command. Samples drawn from these resized images effectively reduce the sampling resolution and therefore the frequencies of features detected. The sample resolution is denoted as arcmin/sample on the rectangular sampling grid of each patch. Limiting the patch size constrains the ICA algorithm to local interactions between image samples and reduces the amount of memory and computation time required for the analysis. The components learned using ICA are generally smaller than the patch size, as shown in Fig. 12. Altering the sample resolution in this way allows us to capture similarities and difference across different image scales; previously we have shown that this method is capable of capturing differences in the spread of position disparities across scale when samples are taken from across the whole image^28^. Here we apply this same technique to study the effect of eccentricity on disparity distributions.

Examples of the components generated by the ICA algorithm are shown in Fig. 12.

### Analysis of binocular components using Gabor functions

Binocularly tuned cells in the visual cortex tend to have receptive field structures that are highly tuned to orientation, frequency, phase and location. Previous research (e.g. by Prince *et al*.) studying the distribution of receptive fields in the macaque visual cortex found that Gabor functions to be a good fit to their empirical data^47^. Gabor functions have the added benefit of providing a parametric function that aids estimation phase and frequency of the carrier signal in noisy data.

From Fig. 12 it is clear that many of the components resemble Gabor functions, with one Gabor function for each eye. We fitted a Gabor function individually to both left and right parts of the components.

The 2D Gabor function is defined as:

3 $$\begin{array}{c}g(x,y:\theta ,f,\varphi ,{\sigma }_{w},{\sigma }_{h},\psi )=w(x,y,{\sigma }_{w},{\sigma }_{h},\psi )c(x,y,f,\varphi ,\theta )={e}^{(-\frac{{\dot{x}}^{2}}{2{\sigma }_{w}^{2}}-\frac{{\dot{y}}^{2}}{2{\sigma }_{h}^{2}})}cos(2\pi zf+\varphi )\\ \dot{x}=xcos\psi -ysin\psi ,\quad \quad \dot{y}=xsin\psi +ycos\psi ,\quad \quad z=xcos\theta -ysin\theta \end{array}$$

where *x* and *y* are pixel locations within the patches. The windowing function w describes a two-dimensional Gaussian window of width *σ*_*w*_, height *σ*_*h*_ and orientation *ψ*. The carrier wave function *c* describes a one-dimensional sinusoid extended to two-dimensions, of frequency *f* phase *ϕ* and orientation *θ*. All the Gabor functions are centred at 0 and generated over a two dimensional image *x* = [(−*n*)/2:*n*/2] and *y* = [(−*n*)/2:*n*/2], where *n* is the size of the component patch. Not all components admitted a successful fit of a Gabor function resulting in rejection of ill fitted components. The exact proportions of rejected components varied according to sampling resolution and sampling location, and are described in the results section. A complete description of the fitting method and analysis of fitting accuracy pass been previously published by^28^.

### Analysis of disparity

Disparity tuning was assessed by comparing the parameters of the Gabor functions fitted to the left and right parts of each component. Two measures were of particular interest: position disparity – the separation of the windowing function, and phase disparity – the difference in phase between the two Gabor functions when phase is measured relative to the centre of the windowing function.

### Data-set

Binocular photographic image data and (Matlab) source code associated with this publication is available from GitHub at https://github.com/DavidWilliamHunter/Bivis.

## Electronic supplementary material

Dataset 1
